# c-Diadem: a constrained dual-input deep learning model to identify novel biomarkers in Alzheimer’s disease

**DOI:** 10.1186/s12920-023-01675-9

**Published:** 2023-10-13

**Authors:** Sherlyn Jemimah, Aamna AlShehhi, for the Alzheimer’s Disease Neuroimaging Initiative

**Affiliations:** Department of Biomedical Engineering, Khalifa University, PO Box 127788, Abu Dhabi, United Arab Emirates

**Keywords:** Alzheimer’s disease, Biomarkers, Neural network, Deep learning, Binary classification, Genomics, Genetics, Gene expression

## Abstract

**Background:**

Alzheimer’s disease (AD) is an incurable, debilitating neurodegenerative disorder. Current biomarkers for AD diagnosis require expensive neuroimaging or invasive cerebrospinal fluid sampling, thus precluding early detection. Blood-based biomarker discovery in Alzheimer’s can facilitate less-invasive, routine diagnostic tests to aid early intervention. Therefore, we propose “c-Diadem” (constrained dual-input Alzheimer’s disease model), a novel deep learning classifier which incorporates KEGG (Kyoto Encyclopedia of Genes and Genomes) pathway constraints on the input genotyping data to predict disease, i.e., mild cognitive impairment (MCI)/AD or cognitively normal (CN). SHAP (SHapley Additive exPlanations) was used to explain the model and identify novel, potential blood-based genetic markers of MCI/AD.

**Methods:**

We developed a novel constrained deep learning neural network which utilizes SNPs (single nucleotide polymorphisms) and microarray data from ADNI (Alzheimer’s Disease Neuroimaging Initiative) to predict the disease status of participants, i.e., CN or with disease (MCI/AD), and identify potential blood-based biomarkers for diagnosis and intervention. The dataset contains samples from 626 participants, of which 212 are CN (average age 74.6 ± 5.4 years) and 414 patients have MCI/AD (average age 72.7 ± 7.6 years). KEGG pathway information was used to generate constraints applied to the input tensors, thus enhancing the interpretability of the model. SHAP scores were used to identify genes which could potentially serve as biomarkers for diagnosis and targets for drug development.

**Results:**

Our model’s performance, with accuracy of 69% and AUC of 70% in the test dataset, is superior to previous models. The SHAP scores show that SNPs in PRKCZ, PLCB1 and ITPR2 as well as expression of HLA-DQB1, EIF1AY, HLA-DQA1, and ZFP57 have more impact on model predictions.

**Conclusions:**

In addition to predicting MCI/AD, our model has been interrogated for potential genetic biomarkers using SHAP. From our analysis, we have identified blood-based genetic markers related to Ca^2+^ ion release in affected regions of the brain, as well as depression. The findings from our study provides insights into disease mechanisms, and can facilitate innovation in less-invasive, cost-effective diagnostics. To the best of our knowledge, our model is the first to use pathway constraints in a multimodal neural network to identify potential genetic markers for AD.

## Background

Alzheimer’s disease (AD) is the most common form of dementia, characterized by a gradual loss of cognition and memory. AD is expected to affect around 78 million older adults by 2030 [1]. Diagnosis of probable or possible AD is based on symptom presentation and neuropsychological testing according to NINCDS-ADRDA (National Institute of Neurological and Communicative Disorders and Stroke and the Alzheimer’s Disease and Related Diseases Association) criteria [2]. Although neuroimaging and biofluid-based biomarkers exist for diagnosis in living patients, they are invasive, not widely accessible and not amenable for definitive diagnosis [3]. On the other hand, blood tests are less invasive, facilitate screening and early diagnosis, and confer significant cost benefits [3]. Therefore, blood-based biomarker discovery has become a key area of clinical research in AD [4].

Disease-related genes are commonly identified using genome-wide association studies (GWAS) [5], in which several million single nucleotide polymorphisms (SNPs) are tested for association with a specific trait or disease. While APOE ε4 has been established as a significant risk factor for AD susceptibility, AD is considered a complex trait in which a combination of genetic and environmental factors influences disease pathology [5]. Several genes, including CR1, PICALM, ABCA7, HLA-DRB5/HLA-DRB1, and SLC24A4/RIN3 have been identified as AD susceptibility loci by GWAS, and are associated with inflammation, immune response, lipid metabolism and intracellular trafficking [5]. Nevertheless, AD-associated variants (except APOE ε4) have small effect sizes and are not sufficient to explain a large component of genetic heritability, which is estimated to contribute up to 80% of the observed disease phenotype [6].

Machine learning (ML) methods have greater power to capture interactions between single variants and genes, which may explain heritability to a greater extent. For instance, Segura et al. [7] showed that tree-based methods prioritized SNPs located in genes PVRL2, TOMM40, APOE, and APOC1. Genomic profiles showed interactions between specific SNPs in both UK BioBank and the Alzheimer’s Disease Neuroimaging Initiative (ADNI) datasets. The telescopic ML-based GWAS strategy proposed by Squillario et al. [8] integrated results at SNP, gene, and pathway levels of information. The authors identified TOMM40 and GRM7 as strongly associated with APOE ε4 status at the SNP, gene and pathway levels, thus providing support for cumulative polygenetic susceptibility to AD. Given that AD pathology is associated with systemic changes reflected in other parts of the body [9–11], we hypothesize that ML models are better suited to identify blood-based markers for AD.

Furthermore, recently published artificial intelligence (AI) models have focussed on interpretability, such as the deep learning framework proposed by Qui et al. [12], which provides a disease probability map as an intermediate output after training a fully convolutional network on magnetic resonance imaging (MRI) data. The disease probability map is fed into a multilayer perceptron (MLP) for binary classification along with clinical information such as age, gender and mini-mental state examination (MMSE) scores. Deep learning models in cancer have incorporated biological information in the form of pathway constraints from KEGG (Kyoto Encyclopedia of Genes and Genomes) [13] and Reactome [14] to increase prediction performance and enhance interpretability. Moreover, several studies [15–18] have employed SHapley Additive exPlanations (SHAP) [19], to understand model prediction and derive insights into disease markers and pathology.

Therefore, in our study, we present c-Diadem, a constrained dual-input AI model for Alzheimer’s disease which incorporates KEGG pathway constraints to accurately predict disease status. We used genomic and expression data from the Alzheimer’s Disease Neuroimaging Inititative (ADNI) [20] for training, validation, and testing. We further explain and interpret the model using SHAP to identify novel, potential genetic markers of AD.

## Methods

**Fig. 1:**
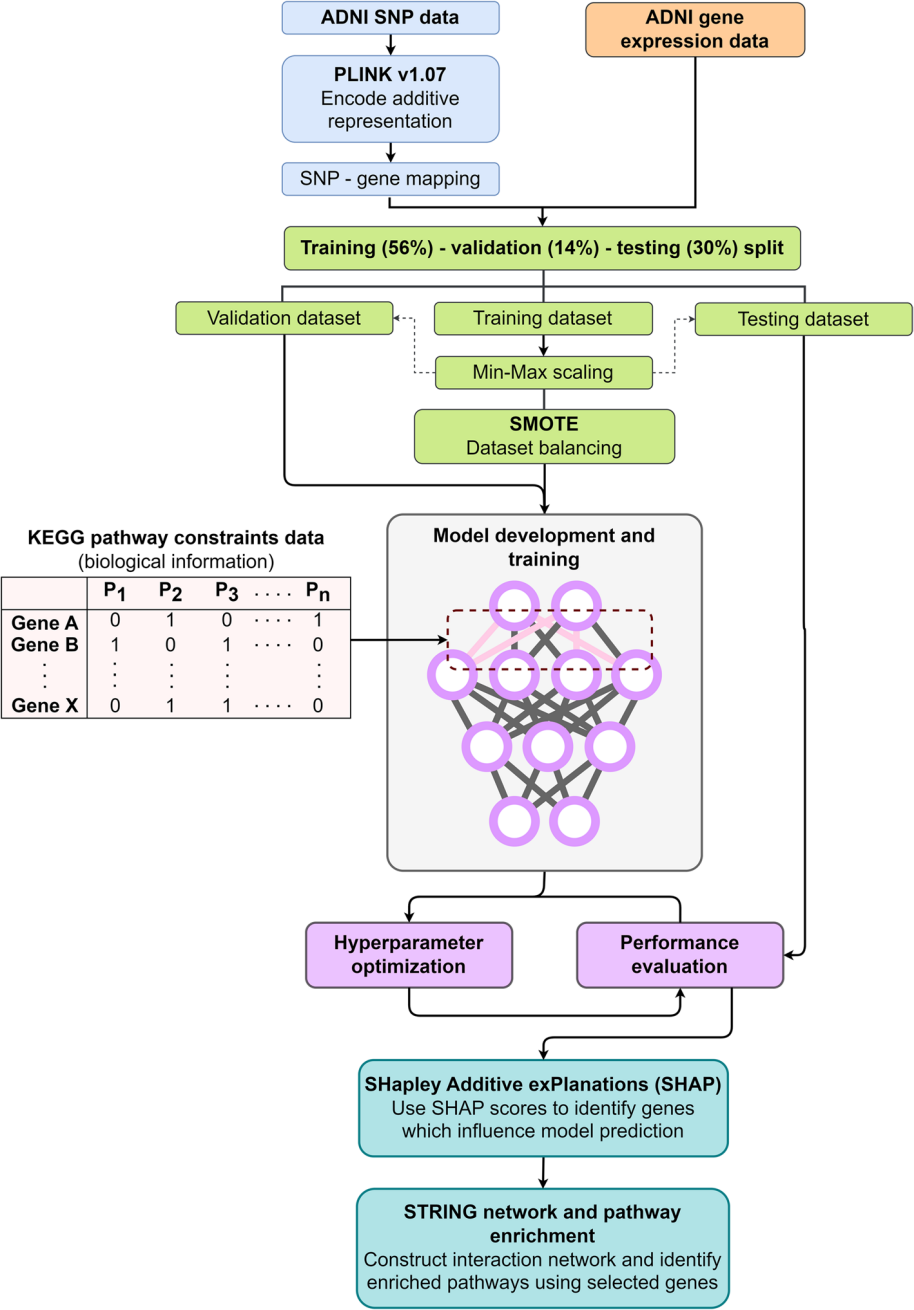
Flow diagram showing an overview of data preprocessing, model development and interpretation

We have summarized our data preprocessing, model development and interpretation steps in a flow diagram in Fig. 1. The datasets and methods have been described in detail in the following sections.

### Alzheimer’s Disease Neuroimaging Initiative (ADNI)

Data used in the preparation of this article were obtained from the Alzheimer’s Disease Neuroimaging Initiative (ADNI) database (adni.loni.usc.edu). The ADNI was launched in 2003 as a public–private partnership, led by Principal Investigator Michael W. Weiner, MD. The primary goal of ADNI has been to test whether serial magnetic resonance imaging (MRI), positron emission tomography (PET), other biological markers, and clinical and neuropsychological assessment can be combined to measure the progression of mild cognitive impairment (MCI) and early AD. For up-to-date information, see www.adni-info.org. In addition to MRI and PET neuroimaging of patients at regular intervals, ADNI has collected and analyzed whole blood samples for genotyping and gene expression analysis. Table 1 provides a summary of the genotyping data provided by ADNI. Blood gene expression profiling was conducted using Affymetrix Human Genome U219 Array for 744 samples in the ADNI2 and ADNI-GO (ADNI-Grand Opportunity) phases [20].

**Table 1:** ADNI genotyping data summary

Phase	Platform	Variants	Participants	Genome Assembly	DbSNP Build
ADNI1	Illumina Human 610-Quad BeadChip	620901 SNP and CNV markers	757	hg18	129
ADNIGO/ADNI2	Illumina Human OmniExpress BeadChip	730525 SNPs and CNV markers	793	hg18	129
ADNI3	Illumina Omni 2.5 M (WGS Platform)	759993 SNPs and CNV markers	327	hg38	155

### Data preprocessing

Genotyping data for ADNI1, ADNI2/ADNIGO and ADNI3 are available for 1877 participants in total. Out of 1877 participants, 626 participants from the ADNIGO/ADNI2 phase have also provided whole blood samples for the gene expression assay. Thus, these 626 patients were selected for our study and their genotyping data was used as the genomic data source for our model. All SNPs have been identified using the hg18 build and therefore, the input data does not contain any missing information. SNP data for the selected patients were downloaded in PLINK binary format, consisting of.bed,.bim and.fam files. The.bed file is the primary representation of genotype calls of biallelic variants. The.bim file accompanies the.bed file and provides extended variant information, i.e., SNP IDs, base-pair coordinates, and the minor and major alleles. The.fam file provides sample information, including parent IDs and phenotype. After minor allele frequency (MAF) filtering, we utilized the.bim file to identify the minor allele and encoded SNPs using additive representation (i.e., 0 = homozygous dominant, 1 = heterozygous, 2 = homozygous recessive). The dbSNP ID was used as the unique identifier for SNPs. Then, coding SNPs (which occur in coding sequences, or CDS) were mapped to their corresponding gene loci. Input values represent the aggregate additive value of all SNPs mapped to the coding sequences of individual genes. The gene expression data merged with the SNP data matrix represents model inputs. For the development of a constrained model, KEGG pathway data was used to populate the constraints matrix.

We applied a train/test/validation split of 56–30-14%. The dataset consists of 212 CN, 317 MCI and 97 AD samples. Thus, the datasets were balanced using target stratification and the Synthetic Minority Oversampling Technique (SMOTE). SMOTE boosted model performance compared to other oversampling techniques such as ADASYN (adaptive synthetic), SVMSMOTE (support vector machines SMOTE) and borderline SMOTE (oversampling limited to borderline cases). Moreover, SMOTE has been used previously in predicting Alzheimer’s disease using MRI images [21]. Therefore, SMOTE was the preferred strategy for addressing data imbalance in our study. Finally, sample labels were converted to 2 × 1 scalars using one-hot encoding.

### AI model design

We used Python v3.8 with the Functional API of keras v2.4.3 to design and build the constrained and unconstrained neural networks. The constrained model topology is shown in Fig. 1a. The input data for our model includes genotyping data for 5188 mapped genes and gene expression data for 19,403 genes. The SNP data and gene expression data were provided as separate inputs. The edges between the genotyping input layer and the pathway layers were constrained using prior biological information from KEGG pathway datasets [22]. KEGG data from the Molecular Signatures database (MSigDB) [23] was used to create constraints encoded as a binary weights matrix which sets all non-existent connections among the genes and pathways to zero. Therefore, the edges from the input genes to the unrelated pathways were set to 0 and remained constant during training. The genes were mapped to 186 KEGG pathways, which form the second layer of the constrained model. Then, the pathway features were transformed using a 1D convolutional layer (kernel size 12) before being concatenated with the gene expression input. The concatenated data was passed through a batch normalization layer and three hidden layers. The output layer contains two nodes representing the binary outcomes of CN and MCI/AD. Softmax activation was used to convert the output into the respective CN and MCI/AD probabilities, with the higher probability used for classification of disease status. The hyperparameters are summarised in Table 2.

**Table 2:** Model hyperparameters

	**Hyperparameters**	**Value**
Overall model	Number of layers	14
	Loss function	Binary cross-entropy
	Learning rate	0.005
	Optimizer	Adam
	Training epochs	80
	Callbacks	Validation loss
	Batch size	32
Genotyping input layers	Architecture [Layer name (output shape)]	Input layer (5188) Pathways layer (186) Reshape layer (186, 1) 1D Convolutional layer (186, 12) Flatten layer (2232) Dense layer (150)
Gene expression input layers	Architecture [Layer name (output shape)]	Input layer (19403) Dense layer (150)
Concatenation layer	Output nodes	300
Batch normalization layer	Momentum	0.99
	Epsilon	0.001
Hidden layers	Number of layers	3
	Architecture [Layer name (output shape)]	Dense layer 1 (180) Dense layer 2 (30) Dense layer 3 (15)
Output layer	Output nodes	2
	Activation	Softmax

### Performance evaluation

The predictive performance of the model was evaluated on the validation and test sets using area under the curve (AUC), accuracy, precision, recall and F1 score. The best-performing weights were chosen using a callback on validation loss. The AUC, accuracy, precision, and recall were calculated for each epoch, for both validation and training. The formulae for the performance metrics are provided below.

$$\begin{array}{c}Accuracy=\frac{TP+TN}{TP+FN+TN+FP}\\\begin{array}{c}Precision=\frac{TN}{TN+FP}\\Recall=\frac{TP}{TP+FN}\\F1score=2\cdot\frac{Precision\cdot Sensitivity}{Precision+Sensitivity}\end{array}\end{array}$$

Here, true positives (TP) refer to the number of correctly predicted MCI/AD cases. True negatives (TN) refer to the number of correctly predicted CN cases. False positives (FP) refer to the CN cases incorrectly predicted as MCI/AD. False negatives (FN) refer to MCI/AD cases incorrectly predicted as CN.

### Model interpretation with SHAP

Model interpretation is essential to gain user trust and overcome the ‘black box’ reputation of deep learning models. Lundberg and Lee [19] proposed SHAP values as a unified measure of feature importance, computed using game theory. SHAP scores were computed using the Python shap package (v0.39.0) to identify genes which could potentially be considered biomarkers for diagnosis with prodromal and advanced Alzheimer’s. The top twenty selected genes were then analyzed using STRING (Search Tool for the Retrieval of Interacting Genes/Proteins) [24] to identify enriched pathways and their role in Alzheimer’s disease.

### Statistical analysis

Differences in clinical features between CN and MCI/AD subjects were analyzed using statistical tests for significance. We performed the analysis of variance (ANOVA) test on the age of onset and years of education. We used the Chi-square test for differences in the proportion of male and female participants and the presence of the APOE ε4 allele. Student’s t-tests were utilized for the neuropsychological test scores, namely the MMSE (Mini-Mental State Examination) and CDRSB (Clinical Dementia Rating – Sum of Boxes) scores.

## Results

We developed a constrained deep learning binary classifier which was trained and tested on genotyping and gene expression data from 626 ADNI participants, with a train-test-validation split of 56–30-14%. The data was imbalanced with 212 CN participants and 414 patients with MCI/AD. Therefore, we applied target stratification and SMOTE to balance the training dataset. The inputs for our model include 5188 genes mapped from SNPs and gene expression data for 19,403 genes.

### Clinical cohort characteristics

The clinical characteristics of the CN and MCI/AD are summarized in Table 3. The CN and MCI/AD groups differ significantly in terms of mean age (*p* = 0.001) and proportion of female participants (*p* = 0.015). Both CN and MCI/AD groups have similar years of education. We also observe a higher proportion of MCI/AD patients with the APOE ε4 allele compared to CN subjects (*p* < 0.001). As expected, MCI/AD patients show significantly worse performance in neuropsychological tests such as CDRSB (*p* < 0.001) and MMSE (*p* < 0.001).

**Table 3:** Clinical cohort characteristics

	**CN**	**MCI/AD**	***P*-value**
Number of patients	212	414	
APOE ε4 allele present	58	231	< 0.001
Gender (female %)	105 (49.5%)	163 (39.2%)	0.015
Age	74.6 ± 5.4	72.7 ± 7.6	0.001
Years of education	16.2 ± 2.7	15.9 ± 2.7	0.258
Clinical Dementia Rating – Sum of Boxes	0.07 ± 0.3	2.5 ± 2.5	< 0.001
Mini-Mental State Examination score	29.1 ± 1.2	26.4 ± 4.0	< 0.001

### Model development

**Fig. 2:**
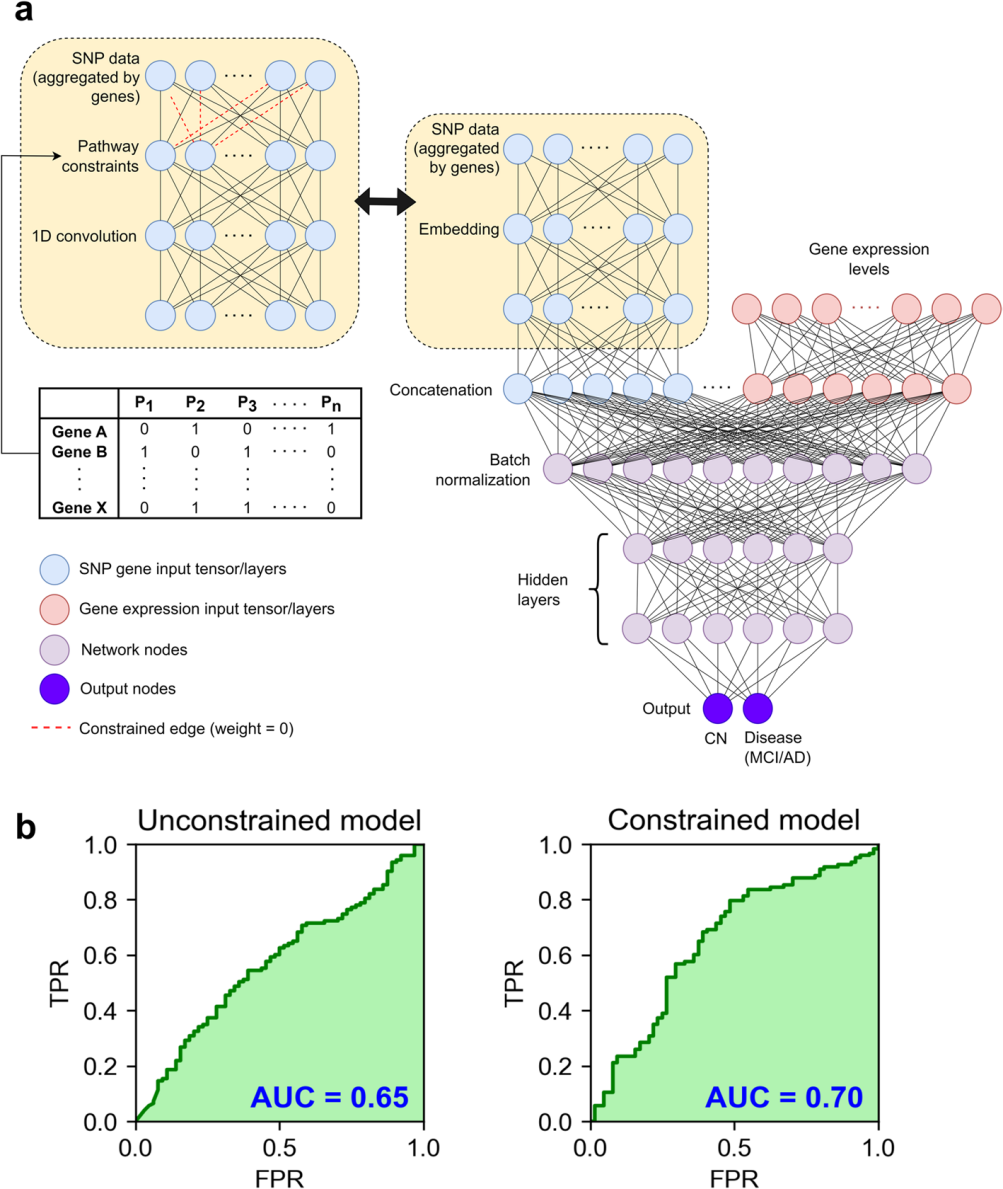
Model topology and performance. **a** Model topology for unconstrained and constrained model. Pathway constraints are used for the constrained model on the SNP input data. **b**)Receiver-operating characteristic (ROC) curves show increased performance (area under the curve (AUC) = 0.70) for constrained model. TPR and FPR stand for True Positive Rate and False Positive Rate respectively

The constrained deep learning model is a parsimonious feed-forward neural network with 14 layers, which uses SNPs and microarray data from ADNI. The topology of the model is depicted in Fig. 2a. The model was used to predict whether a given patient was cognitively normal (CN) or was in the AD spectrum (either the prodromal stage of MCI or advanced AD). The SNP inputs were constrained using KEGG pathway information before concatenation with gene expression data and passed through dense, hidden layers.

The model was trained with the early stopping callbacks on validation loss monitoring to prevent overfitting. The maximum number of epochs was set at 80 with a default batch size of 32. We also developed an unconstrained network with 9 layers, including an embedding layer of size (186, 3), depicted in Fig. 2a, for comparison.

### Model performance

We evaluated the ability of the constrained model to classify the patients as MCI/AD or CN. Our model, c-Diadem, achieved an accuracy of 69% and an AUC of 70% on the test dataset. The inclusion of KEGG pathway constraints increased model accuracy and AUC, as shown in Fig. 2b. The performance metrics of our model have been compared with other classifiers in Table 4. Our model shows a high F1 score of 0.69 compared to the SNP-only deep model (F1 score = 0.53). The F1 score includes both the sensitivity and specificity of the model. With SNPs data, our model shows an accuracy of 0.64 and an AUC of 0.67 (F1 score = 0.64). The accuracy and AUC of the model improve with the inclusion of gene expression data. Therefore, combining SNPs and gene expression inputs helps our model significantly outperform previous models (accuracy = 0.69, AUC = 0.70, F1 score = 0.69). An AUC cut-off of 0.70 or more indicates a moderate-to-high predictive ability for models of dementia risk [25].

**Table 4:** c-Diadem performance metrics compared with current models

Model	Classification Type	Inputs	Evaluation dataset	Accuracy	AUC	F1 score	Reference
c-Diadem	Binary (CN, MCI/AD)	SNPs and gene expression data	ADNI test dataset (30%)	0.6898	0.7027	0.6898	This work
Unconstrained model				0.5935	0.6549	0.5935	This work
c-Diadem		SNPs only		0.6417	0.6702	0.6417	This work
DNN with DEG	Binary (CN, AD)	Blood gene expression	Internal fivefold CV	NA	0.6568	NA	[26]
SNP (deep model)	Binary (CN, MCI/AD)	SNPs	ADNI test set (10%)	0.66	NA	0.53	[27]
RPART	Binary (CN, AD)	SNPs	ADNI validation dataset	0.754	0.614	0.392	[28]

*Abbreviations*: *DNN* Deep neural network, *DEG* Differentially expressed genes, *NA* Not available, *RPART* Recursive Partitioning and Regression Trees

### Feature importance using SHAP

**Fig. 3:**
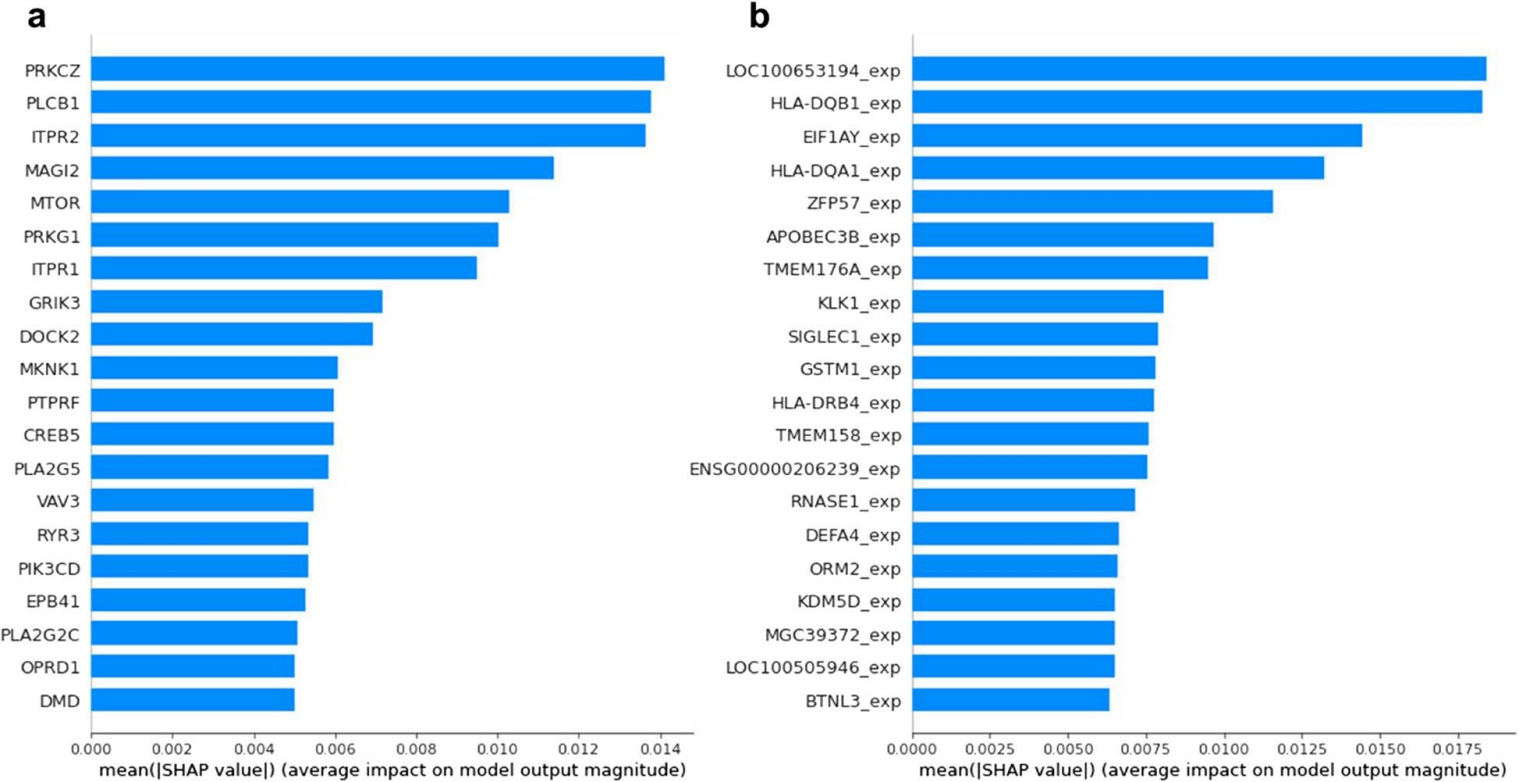
SHAP results based on ADNI (**a**) genotyping and (**b**) gene expression data, computed using the constrained model

To determine the relative importance of genetic features (genotyping and gene expression data), we computed SHAP values in the constrained model for the respective inputs. Figure 3 provides SHAP values for the top twenty features which have the highest impact on model classification. Our results show that SNPs in PRKCZ, PLCB1 and ITPR2 are considered important for prediction of disease status (both MCI and AD). On the other hand, the expression of HLA-DQB1, EIF1AY, HLA-DQA1, and ZFP57 has more predictive value compared to the expression of other genes. The genes selected by SHAP may be considered as potential biomarkers.

**Fig. 4:**
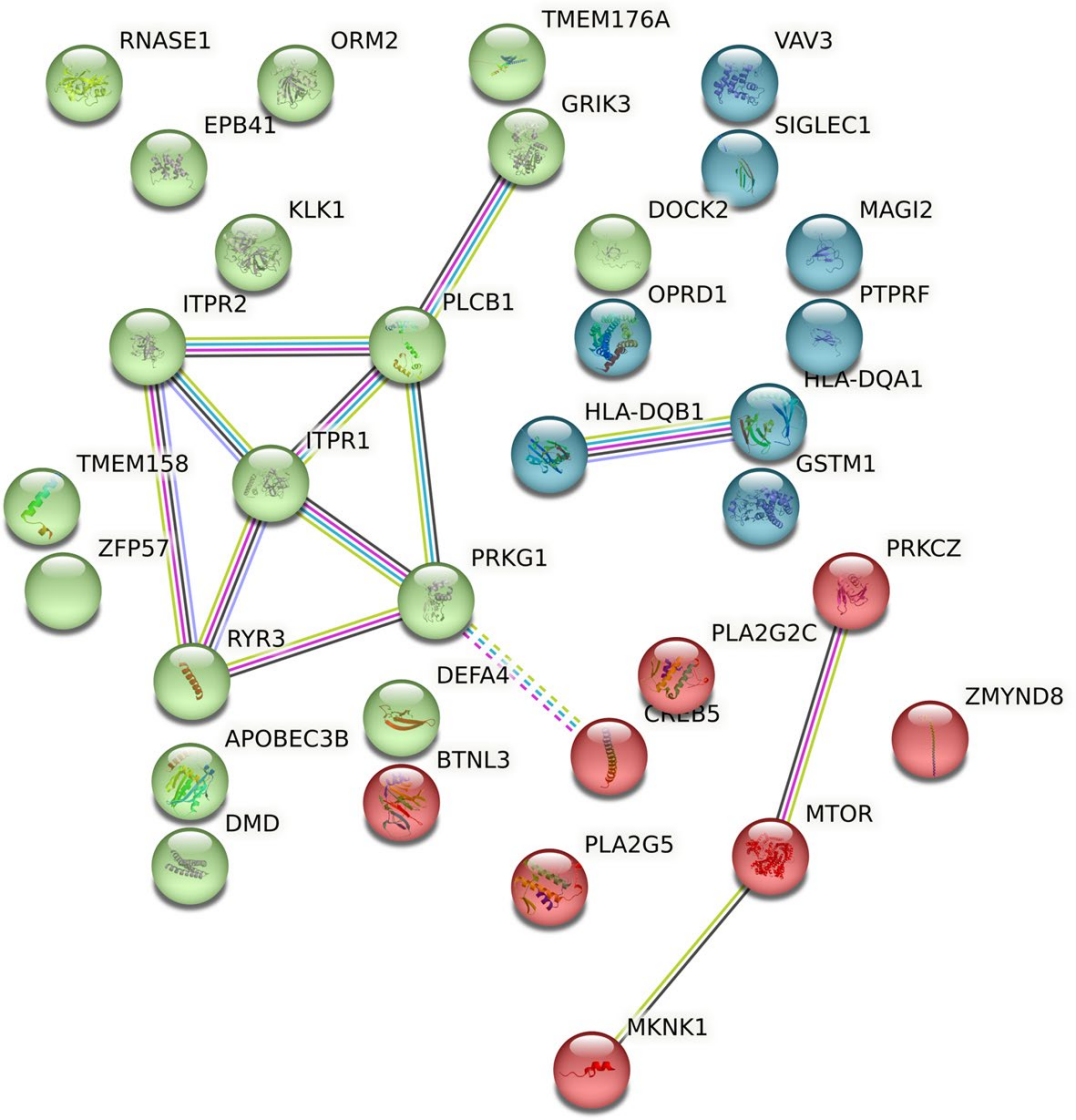
STRING network showing colored clusters

We analyzed the interaction network formed by SHAP-identified genes using the STRING database. From a total of 40 genes, 34 genes were used to construct the interaction network. Some genes did not have a representative protein and so were not included in the network (LOC100653194, EIF1AY, HLA-DRB4, ENSG00000206239, MGC39372, LOC100505946). The resulting network had significantly more edges than expected (protein–protein interactions (PPI) enrichment *p*-value = 0.00247) which indicates biological relationships among the genes. The network was further clustered into subnetworks using k-means clustering. The subnetwork with the most significant enrichment (*p* = 5.56 × 10^–6^) was associated with pathways involved in the release of Ca^2+^ ions into the cytosol (*p* = 0.0016), as well as pathways commonly associated with long-term depression (false discovery rate (FDR) = 3.77 × 10^–5^) and salivary secretion (FDR = 4.55 × 10^–6^). The results of the pathway enrichment analysis have been as summarized in Table 5. The interaction network with clusters (colored red, green and blue) is represented in Fig. 4.

**Table 5:** Pathway enrichment for gene markers by SHAP

ID	Description	Observed gene count	Background gene count	Strength	FDR	Matched genes
***Gene Ontology (GO) Biological Process***						
GO:0051282	Regulation of sequestering of calcium ion	5	134	1.63	0.0012	ITPR1,PLCB1,DMD,ITPR2,RYR3
GO:0051209	Release of sequestered calcium ion into cytosol	4	61	1.88	0.0016	ITPR1,PLCB1,ITPR2,RYR3
GO:0044057	Regulation of system process	6	592	1.07	0.0135	ITPR1,EPB41,DMD,PRKG1,ITPR2,RYR3
GO:0019722	Calcium-mediated signaling	4	165	1.45	0.0167	ITPR1,DMD,ITPR2,RYR3
GO:0019932	Second-messenger-mediated signaling	5	354	1.21	0.0167	ITPR1,DMD,PRKG1,ITPR2,RYR3
GO:1,903,779	Regulation of cardiac conduction	3	68	1.71	0.0359	ITPR1,ITPR2,RYR3
***GO Molecular Functions***						
GO:0015278	Calcium-release channel activity	3	17	2.31	0.002	ITPR1,ITPR2,RYR3
GO:0099094	Ligand-gated cation channel activity	4	101	1.66	0.0023	ITPR1,GRIK3,ITPR2,RYR3
GO:0005220	Inositol 1,4,5-trisphosphate-sensitive calcium-release channel activity	2	3	2.88	0.0033	ITPR1,ITPR2
GO:0035091	Phosphatidylinositol binding	4	252	1.26	0.024	ITPR1,PLCB1,EPB41,ITPR2
GO:0070679	Inositol 1,4,5 trisphosphate binding	2	13	2.25	0.0266	ITPR1,ITPR2
***KEGG Pathways***						
hsa04970	Salivary secretion	5	89	1.81	4.55E-06	ITPR1,PLCB1,PRKG1,ITPR2,RYR3
hsa04730	Long-term depression	4	59	1.89	3.77E-05	ITPR1,PLCB1,PRKG1,ITPR2
hsa04540	Gap junction	4	87	1.72	0.00011	ITPR1,PLCB1,PRKG1,ITPR2
hsa04713	Circadian entrainment	4	92	1.7	0.00011	ITPR1,PLCB1,PRKG1,RYR3
hsa04724	Glutamatergic synapse	4	111	1.62	0.00017	ITPR1,PLCB1,GRIK3,ITPR2
hsa04611	Platelet activation	4	122	1.58	0.0002	ITPR1,PLCB1,PRKG1,ITPR2
hsa04270	Vascular smooth muscle contraction	4	133	1.54	0.00023	ITPR1,PLCB1,PRKG1,ITPR2
hsa04371	Apelin signaling pathway	4	131	1.55	0.00023	ITPR1,PLCB1,ITPR2,RYR3
hsa04720	Long-term potentiation	3	64	1.73	0.00062	ITPR1,PLCB1,ITPR2
hsa04924	Renin secretion	3	66	1.72	0.00062	ITPR1,PLCB1,ITPR2
hsa04927	Cortisol synthesis and secretion	3	65	1.73	0.00062	ITPR1,PLCB1,ITPR2
hsa04929	GnRH secretion	3	63	1.74	0.00062	ITPR1,PLCB1,ITPR2
hsa04918	Thyroid hormone synthesis	3	74	1.67	0.00073	ITPR1,PLCB1,ITPR2
hsa04971	Gastric acid secretion	3	73	1.67	0.00073	ITPR1,PLCB1,ITPR2

## Discussion

Clinical diagnosis through blood sampling would be preferable to cerebrospinal fluid (CSF) sampling, as it is less invasive for patients and amenable for longitudinal monitoring. However, there are several challenges to testing for conventional biomarkers in blood samples. One major issue in blood sampling as a diagnostic tool is the high background of plasma proteins such as albumin and immunoglobulin against which extremely low levels of amyloid-β and tau proteins would need to be quantitated. The low levels of these proteins may be further subject to metabolization and clearing by physiological processes [29]. Moreover, α-synuclein is highly expressed by red blood cells, precluding its use as a blood-based biomarker [30]. Thus, we have implemented a constrained AI model to probe genomic and gene expression data from ADNI for alternative blood-based biomarkers.

Recent studies indicate that the development of Alzheimer’s disease is associated with systemic changes in the neuronal environment reflected in other parts of the body. Studies in peripheral blood mononuclear cells (PBMCs) from amnestic MCI and AD patients show differential expression of senescence markers, such as cell cycle blockade (p16 and p53), DNA damage response (γH2AX) and proinflammatory IL-6/-8 mRNA levels [9]. Garfias et al. [10] have reported significantly higher levels of activated lymphocytes in AD patients. Moreover, a gene expression analysis of PBMC samples in the AddNeuroMed cohort [31] identified DEGs significantly enriched in pathways related to T cell and neutrophil activation in immune response, lymphocyte differentiation, protein serine/threonine kinase activity, GTPase and DNA transcription factor binding [11, 32].

Our findings indicate that the number of SNPs located in ITPR1, PLCB1, DMD, ITPR2 and RYR3 genes are indicative of the dysregulation of processes related to Ca^2+^ sequestration and release. Increased calcium loads in the cytosol can lead to the formation of mitochondrial pore complexes and consequently, apoptosis. Increased Ca^2+^ influx and mitochondrial sequestration is observed in CA1 (cornu ammonis 1) neurons in the hippocampus, which are said to be selectively vulnerable to neurodegeneration in Alzheimer’s disease [33]. The Ca^2+^ influx is facilitated by NR2B overexpression and lower levels of calcium-binding proteins (CBPs) among other factors [34]. Using our model, we found markers of a key event (Ca^2+^ homeostasis dysregulation) in data extracted from patient blood samples. We also observe that pathways related to long-term depression are enriched in our study. Depression is a known risk factor for cognitive decline [35] and up to 50% of AD patients are known to suffer depression [36]. Moreover, from the selected genes, HLA-DQA1 has been reported previously as a risk factor in late-onset Alzheimer’s disease using GWAS [37] and differential expression analysis [38].

It is important to note that our model is limited by the pathway constraints derived from our dataset of choice, KEGG. The genotyping input data of our model has been populated by coding SNPs. Coding SNPs were utilized as they are easily mapped to genes and their respective pathways. Nonetheless, we believe our model’s performance can be enhanced by the inclusion of non-coding SNPs as well as other types of -omics data. We also recognize that the potential genetic biomarkers identified by our study require experimental validation. We hope to explore biomarkers at different stages of Alzheimer’s by refining and training the model to distinguishing between MCI and AD patients in a future study. Refining the model and addressing limitations to enhance performance will help emphasis the validity of our findings.

## Conclusion

The development of alternative biomarkers in Alzheimer’s are foundational for developing less-invasive diagnostics as well as breakthroughs in drug development. Therefore, we have developed a constrained, explainable deep learning model incorporating biological information to accurately predict the disease status of patients. Further, we used SHAP to identify potential biomarkers, which are associated with pathways known to be dysregulated in Alzheimer’s. Therefore, our method can be used to drive research in drug development and diagnostics for Alzheimer’s disease and other dementias.

## Data Availability

The models are made available on GitHub at https://github.com/Sherlyn-J/KU-BMED/. The data that support the findings of this study are available from ADNI, but restrictions apply to the availability of these data, which were used under license for the current study, and so are not publicly available.

## Abbreviations

AD: Alzheimer’s disease
ADNI: Alzheimer’s Disease Neuroimaging Initiative
AUC: Area under the curve
AI: Artificial intelligence
CBPs: Calcium-binding proteins
CSF: Cerebrospinal fluid
CDRSB: Clinical Dementia Rating – Sum of Boxes
CN: Cognitively normal
CA1: Cornu ammonis 1
DNN: Deep neural network
DEG: Differentially expressed genes
FDR: False discovery rate
FPR: False Positive Rate
GO: Gene Ontology
GWAS: Genome-wide association studies
KEGG: Kyoto Encyclopedia of Genes and Genomes
ML: Machine learning
MRI: Magnetic resonance imaging
MCI: Mild cognitive impairment
MMSE: Mini-mental state examination
MSigDB: Molecular Signatures database
MLP: Multilayer perceptron
NINCDS-ADRDA: National Institute of Neurological and Communicative Disorders and Stroke and the Alzheimer’s Disease and Related Diseases Association
NA: Not available
PBMC: Peripheral blood mononuclear cell
PET: Positron emission tomography
PPI: Protein-protein interactions
RF: Random Forest
ROC: Receiver-operating characteristic
STRING: Search Tool for the Retrieval of Interacting Genes/Proteins
SHAP: SHapley Additive exPlanations
SNPs: Single nucleotide polymorphisms
SMOTE: Synthetic Minority Oversampling Technique
TPR: True Positive Rate
